# Construction and Verification of a Frailty Risk Prediction Model for Elderly Patients with Coronary Heart Disease Based on a Machine Learning Algorithm

**DOI:** 10.31083/RCM26225

**Published:** 2025-02-21

**Authors:** Jiao-yu Cao, Li-xiang Zhang, Xiao-juan Zhou

**Affiliations:** ^1^Department of Cardiology, The First Affiliated Hospital of USTC, Division of Life Science and Medicine, University of Science and Technology of China, 230001 Hefei, Anhui, China

**Keywords:** coronary heart disease, elderly, frailty, machine learning, prediction model

## Abstract

**Background:**

This study aimed to develop a machine learning-based predictive model for assessing frailty risk among elderly patients with coronary heart disease (CHD).

**Methods:**

From November 2020 to May 2023, a cohort of 1170 elderly patients diagnosed with CHD were enrolled from the Department of Cardiology of a tier-3 hospital in Anhui Province, China. Participants were randomly divided into a development group and a validation group, each containing 585 patients in a 1:1 ratio. Least absolute shrinkage and selection operator (LASSO) regression was employed in the development group to identify key variables influencing frailty among patients with CHD. These variables informed the creation of a machine learning prediction model, with the most accurate model selected. Predictive accuracy was subsequently evaluated in the validation group through receiver operating characteristic (ROC) curve analysis.

**Results:**

LASSO regression identified the activities of daily living (ADL) score, hemoglobin, low-density lipoprotein cholesterol (LDL-C), total cholesterol (TC), depression, cardiac function classification, cerebrovascular disease, diabetes, solitary living, and age as significant predictors of frailty among elderly patients with CHD in the development group. These variables were incorporated into a logistic regression model and four machine learning models: extreme gradient boosting (XGBoost), random forest (RF), light gradient boosting machine (LightGBM), and adaptive boosting (AdaBoost). AdaBoost demonstrated the highest accuracy in the development group, achieving an area under the ROC curve (AUC) of 0.803 in the validation group, indicating strong predictive capability.

**Conclusions:**

By leveraging key frailty determinants in elderly patients with CHD, the AdaBoost machine learning model developed in this study has shown robust predictive performance through validated indicators and offers a reliable tool for assessing frailty risk in this patient population.

## 1. Introduction

The increasing prevalence of coronary heart disease (CHD) among the elderly aligns with the ongoing trend of population aging, which has introduced a distinct demographic with heightened disease incidence and complex prognoses that substantially affect health outcomes [1]. A comprehensive review highlights the development of over 20 frailty assessment tools, with frailty prevalence among patients with CHD ranging from 10% to 60%, contingent on cardiovascular disease (CVD) burden, chosen assessment tool, and frailty definition cutoffs [2]. Elderly patients with CHD commonly present with multiple comorbidities and reduced cardiac function [3], both of which closely correlate with frailty onset in this age group. Frailty, a multi-dimensional clinical syndrome associated with aging, involves a nonspecific decline in the functioning or physiological reserves of multiple organ systems. Research indicates that frailty prevalence in elderly patients with CHD surpasses that in the general elderly population [4]. This syndrome significantly diminishes quality of life, increasing the risk of adverse hospital events, exacerbating disease progression, and raising mortality risk in elderly patients with CHD [5]. Early detection of frailty and timely intervention in related risk factors can substantially prevent or delay its progression [6].

In recent years, machine learning has been widely applied in the medical field, optimizing predictive accuracy and decision-making, thereby enhancing diagnostics, treatment, and patient care [7]. Studies affirm the value of machine learning in risk prediction, disease diagnosis, and personalized therapeutic strategies [8, 9, 10, 11, 12]. However, specific research on applying machine learning models to predict frailty risk in elderly patients with CHD remains unaddressed. To fill this gap, this study aims to develop a machine learning model for frailty prediction, serving as a practical and efficient screening tool for healthcare providers. Compared to the existing logistic regression model, this study’s machine learning approach offers notable innovations: first, through an in-depth comparison of diverse machine learning algorithms and logistic models, the optimal predictive model is identified, enabling a more comprehensive capture of the complex frailty risk factors and enhancing prediction accuracy. Second, the model leverages sophisticated feature selection and parameter tuning, significantly boosting generalizability and robustness. Moreover, this study emphasizes model interpretability, allowing medical professionals to better comprehend prediction outcomes, thus providing a robust foundation for clinical decision-making.

## 2. Participants and Methods

### 2.1 Research Participants

Between November 2020 and May 2023, a cohort of 1170 elderly patients diagnosed with CHD were recruited from the Department of Cardiology at a tier-3 hospital in Anhui Province, China. Participants were randomized into a development group (585 patients) and a verification group (585 patients) with a 1:1 allocation ratio. Inclusion criteria comprised: (1) meeting the diagnostic standards for CHD [13]; (2) age of 65 years or older; and (3) all study participants provided written informed consent for retrospective data analyses at point of hospital admission before being screened. Exclusion criteria included: (1) patients declining frailty screening; (2) individuals with incomplete clinical data; and (3) cases where frailty status could not be reliably assessed. The study protocol was approved by the Medical Ethics Committee of the First Affiliated Hospital of the University of Science and Technology of China and conformed to the principles outlined in the Declaration of Helsinki.

### 2.2 Data Collection Methods

Data were collected through face-to-face interviews or by consulting the Hospital Information System (HIS) and included: (1) Basic demographic and clinical information, covering gender, age, living situation, smoking and drinking history, body mass index (BMI), and comorbidities such as diabetes, hypertension, cerebrovascular disease, and atrial fibrillation, as well as classification by the New York Heart Association (NYHA). Diabetes criteria included self-reported diagnosis, use of insulin or oral hypoglycemic agents, fasting glucose ≥7 mmol/L, or hemoglobin A1c (HbA1c) ≥6.5% [14]. Hypertension was defined according to the European Society of Hypertension (ESH) Hypertension Guidelines as a systolic blood pressure (SBP) >140 mmHg or a diastolic blood pressure (DBP) ≥90 mmHg [15]. Cerebrovascular disease, encompassing conditions affecting cerebral blood vessels and potentially leading to cognitive impairment or dementia, includes cerebral infarction, transient ischemic attack, cerebral hemorrhage, and cerebral artery atherosclerosis [16]. Atrial fibrillation, a prevalent arrhythmia, was characterized by a disordered fibrillation wave replacing regular atrial electrical activity, resulting in loss of normal atrial rhythm and an irregular, rapid heart rate [17]. (2) Laboratory indices measured included C-reactive protein (CRP) (0–10 mg/L), hemoglobin (130–175 g/L), serum creatinine (31.7–133.0 µmol/L), total cholesterol (TC) (3.25–5.20 mmol/L), triglycerides (TG) (0.56–1.69 mmol/L), high-density lipoprotein cholesterol (HDL-C) (1.2–1.6 mmol/L), and low-density lipoprotein cholesterol (LDL-C) (3.0–3.5 mmol/L). (3) Additional indicators included activities of daily living (ADL), evaluated through the Barthel Index (BI) in its Chinese version. This scale assesses 10 areas: eating, bathing, dressing, urinary and fecal control, toileting, bed and chair transfers, walking, and stair use. Scores were assigned based on the patient’s level of independence in each area, yielding a total score from 0 to 100, with higher scores indicating greater self-care ability [18]. The BI assessment was conducted *via* a HIS-embedded electronic questionnaire. Anxiety and depression levels were measured using the Hospital Anxiety and Depression Scale (HADS) in its Chinese version, comprising two subscales with 7 items each, scored on a 4-point Likert scale (0–3) for a total range of 0–21. Scores exceeding 7 on either subscale indicated the presence of anxiety or depression, respectively [19]. This evaluation was also administered through an HIS-embedded electronic questionnaire. Frailty assessment followed the FRAIL scale, as recommended by the International Academy of Nutrition and Aging (IANA), adapted for the Chinese population. The scale includes five domains: physical fatigue, decreased resistance, reduced mobility, increased susceptibility to illness, and unintentional weight loss. Each domain scores one point, with a total score of 3 or higher indicating frailty [20]. Based on these criteria, the 1170 elderly patients were categorized into a frailty group (402 cases) and a non-frailty group (768 cases).

### 2.3 Statistical Methods

Statistical analyses were conducted using SPSS version 19.0 (IBM Corp., Chicago, IL, USA) and R software version 3.6.1 (R Foundation for Statistical Computing, Vienna, Austria). In SPSS, all patients were randomly allocated to groups using a random number generator, with an initial seed value of 2, resulting in the random assignment of 1170 patients into development and validation groups according to the visualization score box. Descriptive statistics included frequency counts, percentages, means (standard deviation), and medians (25th, 75th percentiles). Group comparisons utilized the Pearson chi-square test for categorical variables, the Mann-Whitney U test for ordinal or non-parametric data, and the independent samples *t*-test for continuous variables, as appropriate. In the development group, variables related to frailty in elderly patients with CHD were analyzed using the least absolute shrinkage and selection operator (LASSO) regression. The predictive performance of the machine learning models was assessed by plotting receiver operating characteristic (ROC) curves for both the development and validation groups and calculating the area under the ROC curve (AUC). Statistical significance was set at a *p*-value of less than 0.05.

## 3. Results

### 3.1 Comparison of Clinical Data between the Development Group and the Verification Group

Participants were randomly allocated into development and validation groups in a 1:1 ratio, with 585 individuals in each group. No significant differences in clinical characteristics were observed between the groups (*p* > 0.05), confirming group homogeneity. Detailed comparisons are provided in Table 1.

**Table 1.** **Comparison of clinical data between the development group and the verification group**.

Variables	Category	Total (n = 1170)	Development group (n = 585)	Verification group (n = 585)	Statistic	*p*-value
Depression, n (%)	No	909 (77.69)	463 (79.15)	446 (76.24)	1.425^a^	0.233
	Yes	261 (22.31)	122 (20.85)	139 (23.76)		
Anxiety, n (%)	No	955 (81.62)	480 (82.05)	475 (81.20)	0.142^a^	0.706
	Yes	215 (18.38)	105 (17.95)	110 (18.80)		
Cardiac function classification, n (%)	Grade I–II	961 (82.14)	478 (81.71)	483 (82.56)	0.146^a^	0.703
	Grade III–IV	209 (17.86)	107 (18.29)	102 (17.44)		
Complicated with atrial fibrillation, n (%)	No	1008 (86.15)	502 (85.81)	506 (86.50)	0.115^a^	0.735
	Yes	162 (13.85)	83 (14.19)	79 (13.50)		
Combined with cerebrovascular disease, n (%)	No	1125 (96.15)	558 (95.38)	567 (96.92)	1.872^a^	0.171
	Yes	45 (3.85)	27 (4.62)	18 (3.08)		
Combined with diabetes, n (%)	No	950 (81.20)	476 (81.37)	474 (81.03)	0.022^a^	0.881
	Yes	220 (18.80)	109 (18.63)	111 (18.97)		
Complicated with hypertension, n (%)	No	914 (78.12)	467 (79.83)	447 (76.41)	2.000^a^	0.157
	Yes	256 (21.88)	118 (20.17)	138 (23.59)		
Living alone, n (%)	No	855 (73.08)	426 (72.82)	429 (73.33)	0.039^a^	0.843
	Yes	315 (26.92)	159 (27.18)	156 (26.67)		
Drinking history, n (%)	No	1014 (86.67)	503 (85.98)	511 (87.35)	0.473^a^	0.491
	Yes	156 (13.33)	82 (14.02)	74 (12.65)		
Smoking history, n (%)	No	932 (79.66)	473 (80.85)	459 (78.46)	1.034^a^	0.309
	Yes	238 (20.34)	112 (19.15)	126 (21.54)		
Frailty, n (%)	No	768 (65.64)	375 (64.10)	393 (67.18)	1.228^a^	0.268
	Yes	402 (34.36)	210 (35.90)	192 (32.82)		
Gender, n (%)	Female	448 (38.29)	230 (39.32)	218 (37.26)	0.521^a^	0.470
	Male	722 (61.71)	355 (60.68)	367 (62.74)		
Age, median (IQR)	/	72.00 (68.00, 75.00)	72.00 (68.00, 75.00)	72.00 (68.00, 75.00)	0.154^b^	0.877
BMI, mean (± SD)	/	23.17 ± 1.94	23.21 ± 1.96	23.12 ± 1.91	0.729^c^	0.466
TC, mean (± SD)	/	4.13 ± 0.88	4.12 ± 0.90	4.15 ± 0.86	–0.689^c^	0.491
TG, median (IQR)	/	1.70 (1.25, 2.15)	1.69 (1.19, 2.10)	1.70 (1.28, 2.17)	–1.360^b^	0.174
HDL-C, mean (± SD)	/	1.90 ± 0.30	1.90 ± 0.30	1.91 ± 0.30	–0.864^c^	0.388
LDLC, median (IQR)	/	1.45 (1.31, 1.61)	1.46 (1.32, 1.62)	1.45 (1.30, 1.59)	1.283^b^	0.200
CRP, median (IQR)	/	5.57 (4.24, 6.94)	5.57 (4.32, 6.84)	5.54 (4.19, 7.01)	0.254^b^	0.799
Serum creatinine, mean (± SD)	/	86.00 ± 22.58	86.21 ± 22.53	85.79 ± 22.63	0.317^c^	0.751
Hemoglobin, mean (± SD)	/	130.50 ± 16.83	129.87 ± 17.23	131.13 ± 16.40	–1.282^c^	0.200
ADL score, median (IQR)	/	72.00 (65.00, 78.00)	72.00 (65.00, 78.00)	72.00 (65.00, 79.00)	–0.705^b^	0.481

Note: a, Pearson chi-square test; b, Mann-Whitney rank sum test; c, Independent sample *t*-test; IQR, inter-quartile range; BMI, body mass index; TC, total cholesterol; TG, triglycerides; HDL-C, high-density lipoprotein cholesterol; LDL-C, low-density lipoprotein cholesterol; CRP, C-reactive protein; ADL, activities of daily living.

### 3.2 Comparison of Clinical Data between the Frailty Group and the Non-Frailty Group in the Development Group

In the development group, a comparison of clinical data between the frailty and non-frailty groups revealed statistically significant differences (*p* < 0.05) across eleven variables: living status (alone), smoking history, depression, cardiac function classification, presence of cerebrovascular disease, diabetes, age, TC, LDL-C, hemoglobin, and ADL score, as detailed in Table 2.

**Table 2.** **Comparison of clinical data between the frailty group and the non-frailty group in the development group**.

Variables	Category	Total (n = 585)	Non-frailty group (n = 375)	Frailty group (n = 210)	Statistic	*p*-value
Gender, n (%)	Male	355 (60.68)	226 (60.27)	129 (61.43)	0.076^a^	0.783
	Female	230 (39.32)	149 (39.73)	81 (38.57)		
Living alone, n (%)	No	426 (72.82)	297 (79.20)	129 (61.43)	21.480^a^	<0.001
	Yes	159 (27.18)	78 (20.80)	81 (38.57)		
Drinking history, n (%)	No	503 (85.98)	323 (86.13)	180 (85.71)	0.020^a^	0.889
	Yes	82 (14.02)	52 (13.87)	30 (14.29)		
Smoking history, n (%)	No	473 (80.85)	292 (77.87)	181 (86.19)	6.025^a^	0.014
	Yes	112 (19.15)	83 (22.13)	29 (13.81)		
Depression, n (%)	No	463 (79.15)	315 (84.00)	148 (70.48)	14.916^a^	<0.001
	Yes	122 (20.85)	60 (16.00)	62 (29.52)		
Anxiety, n (%)	No	480 (82.05)	315 (84.00)	165 (78.57)	2.694^a^	0.101
	Yes	105 (17.95)	60 (16.00)	45 (21.43)		
Cardiac function classification, n (%)	Grade I–II	478 (81.71)	317 (84.53)	161 (76.67)	5.574^a^	0.018
	Grade III–IV	107 (18.29)	58 (15.47)	49 (23.33)		
Complicated with atrial fibrillation, n (%)	No	502 (85.81)	322 (85.87)	180 (85.71)	0.003^a^	0.960
	Yes	83 (14.19)	53 (14.13)	30 (14.29)		
Combined with cerebrovascular disease, n (%)	No	558 (95.38)	375 (100.00)	183 (87.14)	50.550^a^	<0.001
	Yes	27 (4.62)	0 (0.00)	27 (12.86)		
Combined with diabetes, n (%)	No	476 (81.37)	320 (85.33)	156 (74.29)	10.837^a^	<0.001
	Yes	109 (18.63)	55 (14.67)	54 (25.71)		
Complicated with hypertension, n (%)	No	467 (79.83)	297 (79.20)	170 (80.95)	0.257^a^	0.612
	Yes	118 (20.17)	78 (20.80)	40 (19.05)		
Age, median (IQR)	/	72.00 (68.00, 75.00)	72.00 (69.00, 76.00)	71.00 (68.00, 74.00)	2.595^b^	0.009
BMI, mean (± SD)	/	23.21 ± 1.96	23.22 ± 1.74	23.19 ± 2.32	0.152^c^	0.879
TC, mean (± SD)	/	4.12 ± 0.90	4.06 ± 0.78	4.23 ± 1.07	–2.033^c^	0.043
TG, median (IQR)	/	1.69 (1.19, 2.10)	1.67 (1.21, 2.06)	1.74 (1.14, 2.18)	–0.225^b^	0.822
HDL-C, mean (± SD)	/	1.90 ± 0.30	1.89 ± 0.29	1.90 ± 0.30	–0.044^c^	0.965
LDL-C, mean (± SD)	/	1.47 ± 0.23	1.43 ± 0.20	1.55 ± 0.27	–5.319^c^	<0.001
CRP, median (IQR)	/	5.57 (4.32, 6.84)	5.61 (4.34, 6.79)	5.48 (4.04, 6.99)	0.120^b^	0.905
Serum creatinine, mean (± SD)	/	86.21 ± 22.53	85.13 ± 20.82	88.12 ± 25.18	–1.458^c^	0.146
Hemoglobin, median (IQR)	/	130.00 (118.00, 142.00)	133.00 (123.00, 146.00)	121.00 (110.00, 134.00)	7.344^b^	<0.001
ADL score, mean (± SD)	/	71.19 ± 9.86	74.89 ± 7.89	64.58 ± 9.58	13.243^c^	<0.001

Note: a, Pearson chi-square test; b, Mann-Whitney rank sum test; c, Independent sample *t*-test; BMI, body mass index; TC, total cholesterol; TG, triglycerides; HDL-C, high-density lipoprotein cholesterol; LDL-C, low-density lipoprotein cholesterol; CRP, C-reactive protein; ADL, activities of daily living; IQR, inter-quartile range.

### 3.3 Screening of Characteristic Variables that Affect the Frailty of Elderly Patients with Coronary Heart Disease in Development Group

**Fig. 1.**
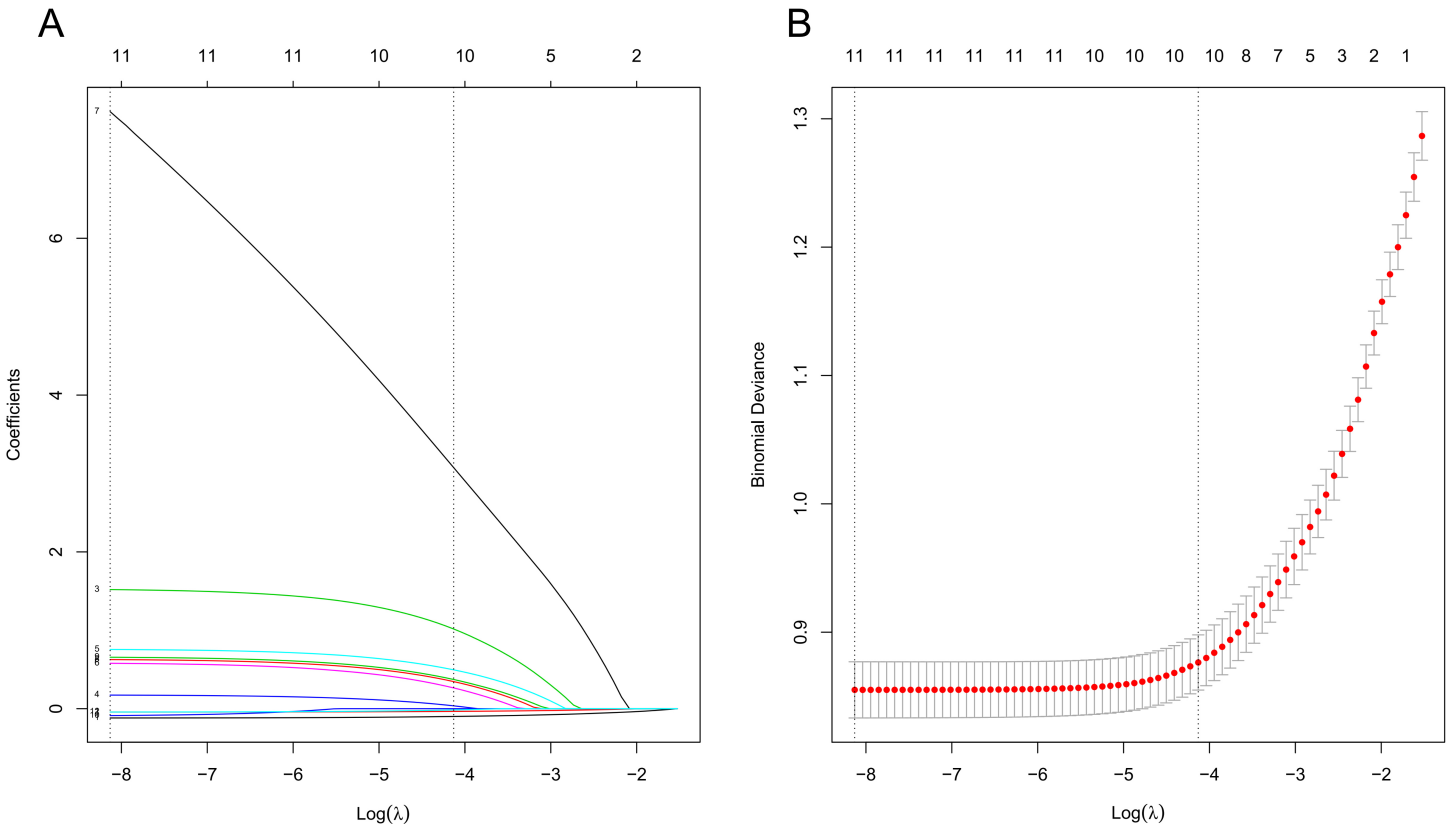
**Results of the LASSO regression analysis**. Note: (A) Path diagram of variable regression coefficient. (B) Cross-validation plot of the LASSO regression analysis. LASSO, least absolute shrinkage and selection operator.

The eleven variables with a *p*-value less than 0.05 identified in Table 2 were included as independent variables, with frailty status in elderly patients with CHD serving as the dependent variable in the development group. LASSO regression analysis, incorporating 10-fold cross-validation, was performed to identify key frailty-related variables. The optimal λ (lambda) value, corresponding to the minimum standard error distance, was determined to be 0.016. At this threshold, the model selected 10 characteristic variables: ADL score, hemoglobin, LDL-C, TC, depression, cardiac function classification, presence of cerebrovascular disease, diabetes, living status (alone), and age, as depicted in Fig. 1.

### 3.4 Establishment and Verification of Machine Learning Prediction Model for the Frailty Risk of Elderly Patients with Coronary Heart Disease

In the development group, frailty risk prediction models were constructed based on the 10 characteristic variables identified by LASSO regression. Five machine learning models were developed: logistic regression, extreme gradient boosting (XGBoost), random forest (RF), light gradient boosting machine (LightGBM), and adaptive boosting (AdaBoost). Internal validation employed 10-fold cross-validation, with a random seed set to 42. Model parameters were as follows: XGBoost: objective (optimization objective function) = binary, learning_rate (learning rate) = 0.3, max_depth (maximum tree depth) = 6, min_child_weight (minimum branch weight sum) = 2, reg_lambda (L2 regularization coefficient) = 1; Logistic regression: C (regularization factor) = 1.0, max_iter (number of iterations) = 100, penalty (regularization type) = l2, tol (convergence metric) = 0.0001; LightGBM: boosting_type (algorithm type) = gbdt, learning_rate (learning rate) = 0.1, max_depth (maximum tree depth) = –1, n_estimators (maximum number of trees) = 100, num_leaves (maximum number of leaves) = 31; RF: criterion (measurement index) = gini, max_depth (maximum tree depth) = None, min_impurity_decrease (minimum branch purity gain) = 0.0, n_estimators (number of trees) = 20; AdaBoost: learning_rate (learning rate) = 1.0, n_estimators (number of single models) = 50.

**Fig. 2.**
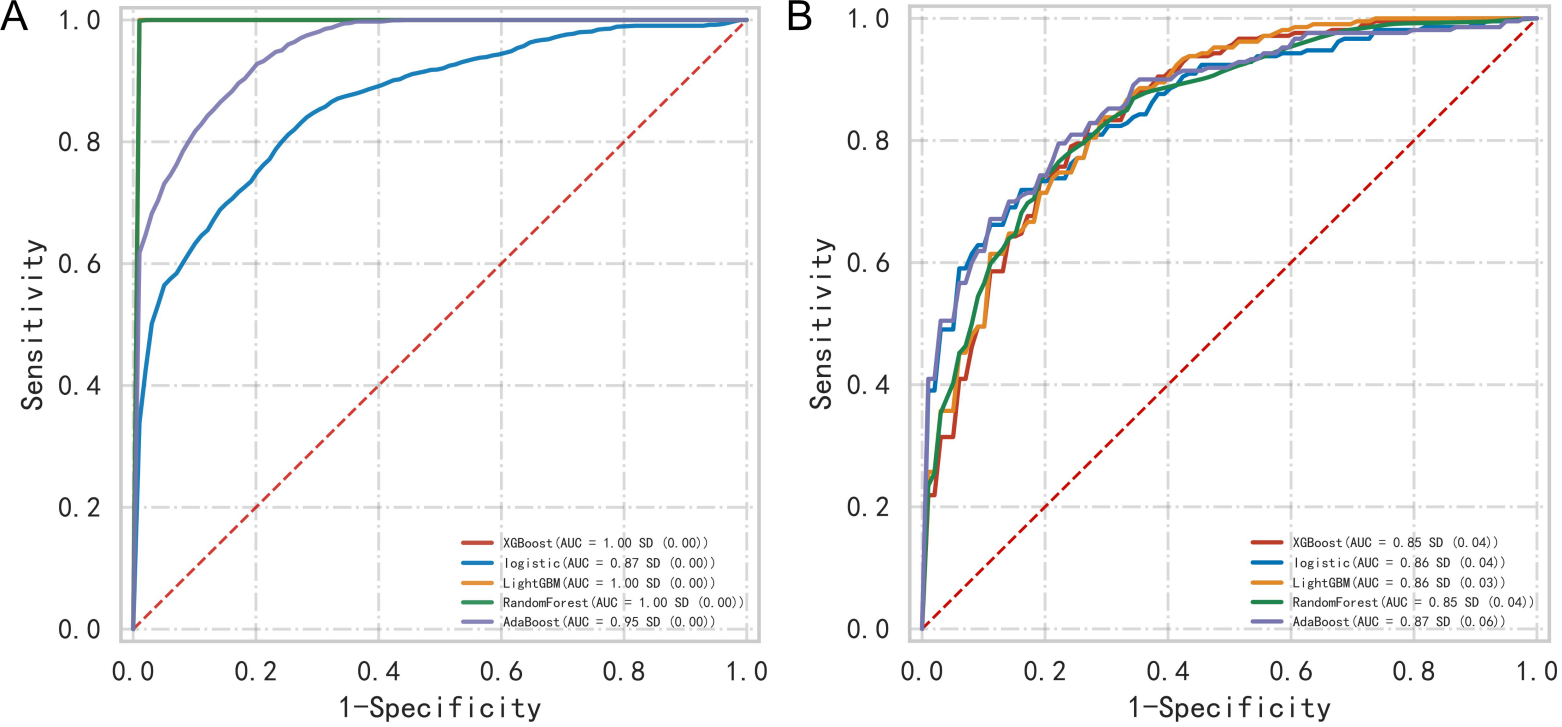
**ROC curves before and after the internal validation of different models in the development group**. Note: (A) ROC curves for different models before internal validation. (B) ROC curves for different models after internal validation. ROC, receiver operating characteristic; AUC, area under the ROC curve; XGBoost, extreme gradient boosting; LightGBM, light gradient boosting machine; AdaBoost, adaptive boosting.

The predictive performance and evaluation metrics for these models are detailed in Table 3. A comparison of the ROC curves across different models is shown in Fig. 2A. To address overfitting risks, all models underwent internal validation using 10-fold cross-validation, with results presented in Table 4. Post-internal validation ROC curve comparisons are depicted in Fig. 2B. Initially, Table 3 indicated that the XGBoost model achieved the highest predictive performance, while Table 4 results suggested AdaBoost exhibited the most stable performance following internal validation. This discrepancy implies potential overfitting in XGBoost, whereas AdaBoost displayed enhanced stability, leading to its selection as the optimal model.

**Table 3.** **Comparison of prediction and evaluation indexes of five machine learning models in the development group**.

Classification model	AUC (SD)	Cutoff (SD)	Accuracy (SD)	Sensitivity (SD)	Specificity (SD)	Positive predictive value (SD)	Negative predictive value (SD)	F1 score (SD)	Kappa (SD)
XGBoost	1.00 (0.00)	0.72 (0.01)	1.00 (0.00)	1.00 (0.00)	1.00 (0.00)	1.00 (0.00)	1.00 (0.00)	1.00 (0.00)	1.00 (0.00)
Logistic	0.87 (0.00)	0.32 (0.05)	0.78 (0.01)	0.81 (0.05)	0.77 (0.05)	0.66 (0.04)	0.88 (0.02)	0.73 (0.01)	0.55 (0.02)
LightGBM	1.00 (0.00)	0.72 (0.02)	1.00 (0.00)	1.00 (0.00)	1.00 (0.00)	1.00 (0.00)	1.00 (0.00)	1.00 (0.00)	1.00 (0.00)
RandomForest	1.00 (0.00)	0.49 (0.03)	1.00 (0.00)	1.00 (0.00)	1.00 (0.00)	1.00 (0.00)	0.99 (0.00)	1.00 (0.00)	0.99 (0.01)
AdaBoost	0.95 (0.00)	0.49 (0.00)	0.86 (0.01)	0.90 (0.03)	0.84 (0.04)	0.76 (0.03)	0.94 (0.02)	0.82 (0.01)	0.71 (0.02)

AUC, area under the ROC curve; XGBoost, extreme gradient boosting; LightGBM, light gradient boosting machine; AdaBoost, adaptive boosting.

**Table 4.** **Comparison of prediction and evaluation indexes of five machine learning models after internal verification in the development group**.

Classification model	AUC (SD)	Cutoff (SD)	Accuracy (SD)	Sensitivity (SD)	Specificity (SD)	Positive predictive value (SD)	Negative predictive value (SD)	F1 score (SD)	Kappa (SD)
XGBoost	0.85 (0.04)	0.83 (0.02)	0.76 (0.04)	0.86 (0.09)	0.75 (0.09)	0.74 (0.05)	0.76 (0.04)	0.80 (0.04)	0.43 (0.11)
Logistic	0.86 (0.04)	0.32 (0.05)	0.76 (0.03)	0.85 (0.09)	0.78 (0.12)	0.63 (0.05)	0.86 (0.05)	0.72 (0.04)	0.49 (0.06)
LightGBM	0.86 (0.03)	0.72 (0.02)	0.78 (0.05)	0.87 (0.09)	0.74 (0.13)	0.77 (0.07)	0.78 (0.05)	0.81 (0.04)	0.47 (0.13)
RandomForest	0.85 (0.04)	0.49 (0.03)	0.79 (0.04)	0.80 (0.10)	0.77 (0.14)	0.74 (0.07)	0.81 (0.04)	0.76 (0.06)	0.52 (0.09)
AdaBoost	0.87 (0.06)	0.49 (0.00)	0.78 (0.04)	0.87 (0.06)	0.78 (0.08)	0.67 (0.05)	0.87 (0.07)	0.75 (0.04)	0.53 (0.09)

AUC, area under the ROC curve; XGBoost, extreme gradient boosting; LightGBM, light gradient boosting machine; AdaBoost, adaptive boosting.

**Fig. 3.**
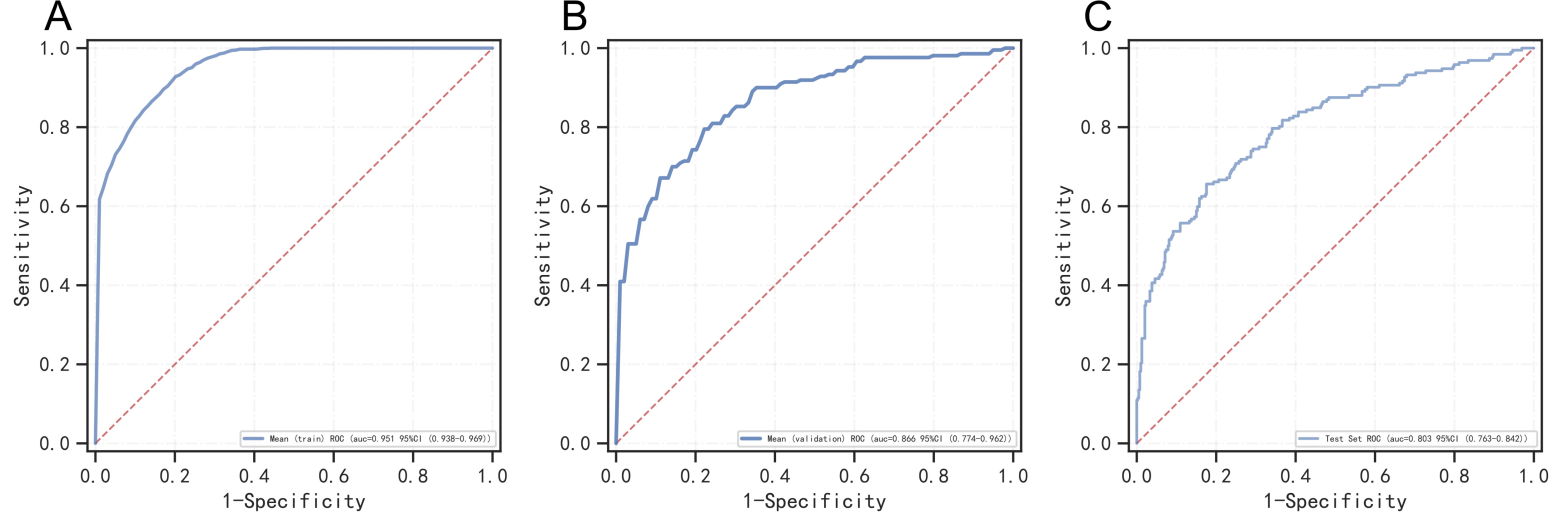
**ROC curve analysis of the AdaBoost model in different data sets**. Note: (A) ROC curve of the AdaBoost model in the development group. (B) ROC curve of the AdaBoost model after internal validation. (C) ROC curve of the AdaBoost model in the verification group. ROC, receiver operating characteristic; AdaBoost, adaptive boosting; CI, confidence interval.

**Fig. 4.**
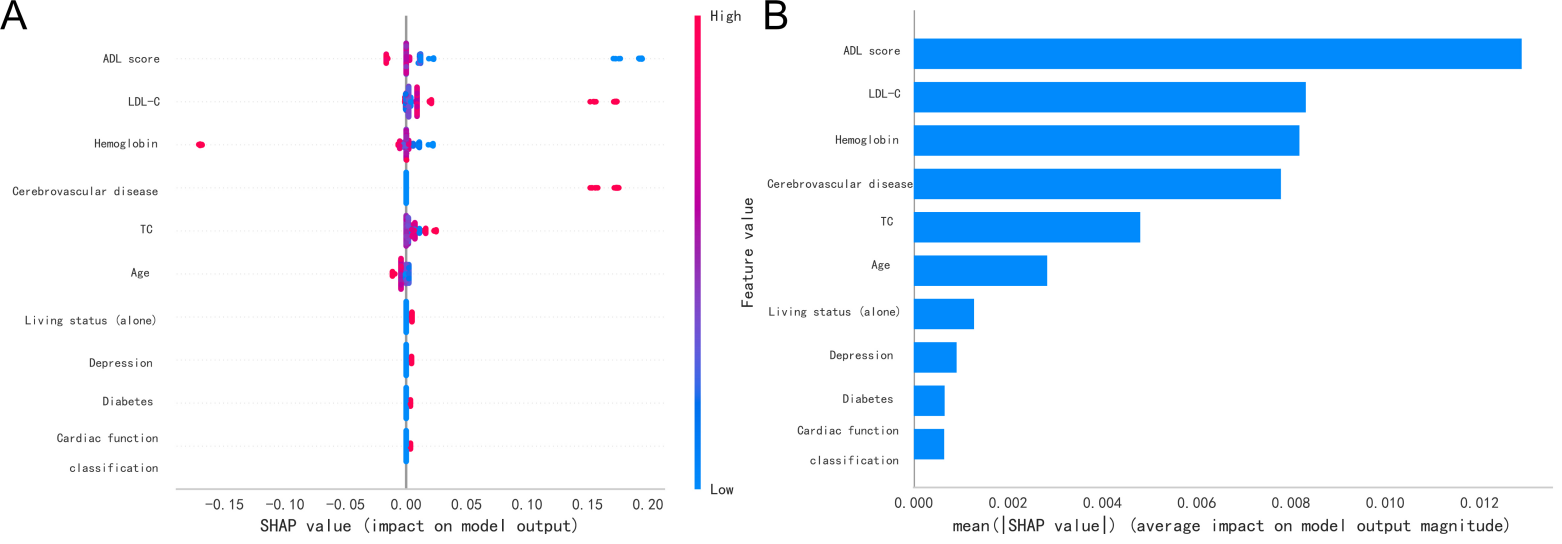
**SHAP value plot for the AdaBoost model**. Note: (A) Summary plot of variable contributions in SHAP analysis. (B) Variable importance ranking plot in SHAP analysis. TC, total cholesterol; LDL-C, low-density lipoprotein cholesterol; ADL, activities of daily living; SHAP, SHapley Additive Explanations.

In the validation group, the AdaBoost model achieved an AUC of 0.803, accuracy of 0.750, sensitivity of 0.66, specificity of 0.79, positive predictive value of 0.61, negative predictive value of 0.83, and an F1 score of 0.63. Given that the AUC in the validation group did not exceed that of the development group by more than 10%, the model is considered well-fitted and demonstrates satisfactory predictive accuracy in the validation cohort. The AdaBoost model’s ROC curves are as follows: Fig. 3A for the development group, Fig. 3B for post-internal validation, and Fig. 3C for the validation group. To evaluate feature importance, SHapley Additive Explanations (SHAP) Value plots were utilized. SHAP analysis clarifies the contribution of each feature to the prediction outcome, as shown in Fig. 4. In Fig. 4A, ADL score, hemoglobin, and age are identified as negative factors for increased frailty risk, whereas LDL-C, TC, depression, cardiac function classification, cerebrovascular disease presence, diabetes, and living status (alone) are positive factors. Fig. 4B highlights the top five variables influencing frailty risk: ADL score, LDL-C, hemoglobin, cerebrovascular disease presence, and TC.

## 4. Discussion

### 4.1 Analysis of Influencing Factors of Frailty in Elderly Patients with Coronary Heart Disease

CHD is a common chronic disease in the elderly. Frailty can accelerate the development of CHD and lead to adverse health outcomes [21]. Understanding the factors influencing frailty in this population is essential for developing a personalized frailty prediction model, which could play a pivotal role in reducing frailty incidence among patients with CHD. This study applied LASSO regression to identify 10 key variables associated with frailty in elderly patients with CHD: ADL score, hemoglobin, LDL-C, TC, depression, cardiac function classification, cerebrovascular disease, diabetes, living status (alone), and age. ADL impairment often indicates muscle mass reduction, a primary mechanism in frailty development [22]. Additionally, older adults with low ADL scores may experience decreased social engagement due to mobility limitations, potentially leading to social isolation and reduced social support—manifestations of social frailty that interact with physiological frailty [23]. Consequently, the severity of ADL impairment directly correlates with frailty risk, with greater impairments indicating higher risk. Anemia, marked by low hemoglobin levels, is also recognized as a significant frailty risk factor among elderly patients [24]. Furthermore, low hemoglobin levels correlate with cognitive decline, particularly in older men, as hemoglobin is essential for oxygen transport throughout the body. Inadequate oxygen delivery due to low hemoglobin can impair muscle function and endurance, thereby increasing frailty susceptibility [25]. Jayanama *et al*. [26]. have identified TC and LDL-C as factors associated with frailty onset, likely due to lipid metabolism and nutritional status, a finding also supported by Yuan *et al*. [27]. Li and Zhao [28] further observed that depression and impaired cardiac function are significant frailty risk factors among the elderly, consistent with this study’s findings. Additionally, Bakhtiari *et al*. [29] reported that frailty correlates with lower levels of preoperative independence, cognitive decline, depressive symptoms, and increased postoperative complications. Another study highlighted a significant association between cerebral microbleeds, particularly in the brainstem, and frailty risk [30]. Burton JK *et al*.’s research [31] indicates that frailty is common in patients presenting with acute stroke and associated with poor outcomes. Bu fan and colleagues [32] also noted a high prevalence of frailty among patients with middle and elderly type 2 diabetes, emphasizing the need for routine frailty assessment and risk factor monitoring, aligning with this study’s outcomes. Furthermore, living alone has been established as an independent risk factor for cognitive decline in elderly individuals [33], and it is recognized as a significant risk factor for frailty [34]. The observed association between age and frailty risk in this study also corroborates prior research findings [35].

### 4.2 The Frailty Risk Prediction Model Based on a Machine Learning Algorithm has Good Prediction Efficiency

XGBoost, a gradient boosting-based ensemble algorithm, enhances predictive accuracy by constructing multiple decision trees, delivering powerful predictive capabilities though requiring substantial computational resources and training time. Logistic regression, a linear classification model suitable for binary classification tasks, is valued for simplicity and interpretability but is limited in handling nonlinear relationships. LightGBM, a gradient boosting decision tree algorithm, expedites training by optimizing the gradient of data instances, providing rapid training and strong predictive performance; however, it can be sensitive to outliers. The RF model, another ensemble algorithm rooted in decision trees, bolsters predictive accuracy by aggregating results from multiple trees, noted for its robustness and predictive strength, though with higher computational demands. AdaBoost, an ensemble algorithm based on weighted voting, boosts predictive performance by adjusting training sample weights, offering robustness to outliers though occasionally susceptible to overfitting. Considering overfitting risks among development group models, internal validation metrics are utilized for model assessment. In terms of AUC, AdaBoost demonstrates the best performance. For accuracy, RF is the leading model; for sensitivity, both LightGBM and AdaBoost perform optimally. Logistic regression and AdaBoost show the highest specificity. LightGBM and AdaBoost excel in positive predictive value and negative predictive value, respectively. For the F1 score, LightGBM is superior, while AdaBoost yields the highest Kappa value.

Considering all evaluation metrics, with AUC as the primary index, this study identifies the AdaBoost algorithm as the most effective machine learning model for frailty prediction. The AdaBoost model achieves an AUC exceeding 0.8 in both the development and validation cohorts, demonstrating strong predictive accuracy. Liu *et al*. [36] similarly examined elderly patients with CHD and developed a nomogram model to predict frailty, reporting a frailty prevalence of 30.07%, comparable to the 34.36% (402/1170) observed in our study. Liu *et al*.’s findings [36] also highlighted factors influencing frailty, such as health status, age, limited social engagement, and impaired daily living activities, consistent with the variables included in our model. However, our study extends this work by incorporating a broader set of predictive indicators. Additionally, unlike the logistic regression-based nomogram, the AdaBoost model offers enhanced classification accuracy and robustness by integrating multiple weak learners, allowing it to better address nonlinear relationships and noise within the data.

The findings from this study underscore the essential role of a frailty assessment in pre-operative evaluations, particularly for patients scheduled for coronary artery bypass grafting (CABG) or other cardiac surgeries. The AdaBoost algorithm, incorporating several critical variables, enables a comprehensive evaluation of frailty in elderly patients with CHD. The ADL score is a key measure of functional status, indicating a patient’s capacity for basic self-care. Hemoglobin levels reflect general health and nutritional status, both of which are significant for recovery and surgical outcomes. LDL-C and TC are key cardiovascular risk markers, where unmanaged levels can exacerbate frailty. Depression, prevalent among elderly patients, impacts both physical health and post-surgical recovery. Cardiac function classification provides insight into disease severity, directly linked to frailty risk. The presence of cerebrovascular disease can further complicate surgery and recovery, contributing to frailty. Diabetes, which impairs healing and elevates surgical risk, is another vital factor. Living status, particularly for patients residing alone, affects available support and post-operative care, potentially impacting frailty. Finally, age remains an inherent risk factor, with older patients facing elevated frailty risks. By leveraging the AdaBoost algorithm to assess these variables, healthcare professionals can better identify individual frailty risks and customize pre-operative interventions accordingly. This personalized approach supports improved surgical outcomes and enhanced quality of life for elderly patients undergoing cardiac procedures.

## 5. Limitations

This study acknowledges several limitations: (1) Data collection was limited to a single tertiary hospital, which may result in a sample that lacks diversity, potentially limiting the generalizability to all elderly patients with CHD. (2) The use of data from a single institution could introduce selection bias, potentially impacting model accuracy and reliability. (3) During feature selection, certain clinically relevant indicators or biomarkers significant for predicting frailty risk may have been inadvertently excluded. (4) The model has not yet been validated on independent external datasets, leaving its performance and stability in external samples uncertain and limiting its external validity. Future research aims to extend collaboration across multiple centers, allowing for validation on independent datasets, with plans to gather a larger, more diverse cohort and incorporate a broader range of indicators to enhance model generalizability and applicability. (5) While patients with coronary artery disease (CAD) may not exhibit overtly disabling symptoms, exertional chest pain remains a critical factor that disrupts daily activities, lowering frailty scores and impacting quality of life. Addressing this limitation is essential for accurately assessing the health status and life quality of patients with CAD. (6) The current prediction model relies primarily on machine learning techniques, which may present a risk of overfitting. To mitigate this, future studies could explore combining multiple algorithms for comprehensive prediction or apply strategies such as regularization, data augmentation, or early stopping to enhance model stability and accuracy.

## 6. Conclusions

In conclusion, the ADL score, hemoglobin levels, LDL-C, TC, presence of depression, cardiac function status, history of cerebrovascular disease, diabetes, living situation (solitary living), and age are identified as key determinants of frailty in elderly patients with CHD. Leveraging these factors, this study developed an AdaBoost machine learning model capable of effectively predicting frailty risk within this patient population.

## Availability of Data and Materials

The datasets used and/or analyzed during the current study are available from the corresponding author on reasonable request.

## References

[1] Hu S, Gao R, Liu L, Zhu M, Wang W, Wang Y (2019). Summary of China Cardiovascular Disease Report 2018. *China Journal of Circulation*.

[2] Afilalo J, Alexander KP, Mack MJ, Maurer MS, Green P, Allen LA (2014). Frailty assessment in the cardiovascular care of older adults. *Journal of the American College of Cardiology*.

[3] Kong D, Chen R, Chen Y, Zhao L, Huang R, Luo L (2024). Bayesian network analysis of factors influencing type 2 diabetes, coronary heart disease, and their comorbidities. *BMC Public Health*.

[4] Qu J, Zhou T, Xue M, Sun H, Shen Y, Liu Y (2021). Relationship Between Medication Literacy and Frailty in Elderly Inpatients With Coronary Heart Disease: A Cross-Sectional Study in China. *Frontiers in Pharmacology*.

[5] Dou Q, Wang W, Wang H, Ma Y, Hai S, Lin X (2019). Prognostic value of frailty in elderly patients with acute coronary syndrome: a systematic review and meta-analysis. *BMC Geriatrics*.

[6] Ng TP, Feng L, Nyunt MSZ, Feng L, Niti M, Tan BY (2015). Nutritional, Physical, Cognitive, and Combination Interventions and Frailty Reversal Among Older Adults: A Randomized Controlled Trial. *The American Journal of Medicine*.

[7] Haug CJ, Drazen JM (2023). Artificial Intelligence and Machine Learning in Clinical Medicine, 2023. *The New England Journal of Medicine*.

[8] Oikonomou EK, Khera R (2023). Machine learning in precision diabetes care and cardiovascular risk prediction. *Cardiovascular Diabetology*.

[9] Wei H, Sun J, Shan W, Xiao W, Wang B, Ma X (2022). Environmental chemical exposure dynamics and machine learning-based prediction of diabetes mellitus. *The Science of The Total Environment*.

[10] Deberneh HM, Kim I (2021). Prediction of Type 2 Diabetes Based on Machine Learning Algorithm. *International Journal of Environmental Research and Public Health*.

[11] Qiao HY, Tang CX, Schoepf UJ, Tesche C, Bayer RR, Giovagnoli DA (2021). Influence of machine learning based on coronary CTA blood flow reserve fraction on treatment decision and clinical outcome of patients with suspected coronary artery disease. *International Journal of Medical Radiology*.

[12] Meng HY, Jin WL, Yan CK, Yang H (2019). The Application of Machine Learning Techniques in Clinical Drug Therapy. *Current Computer-Aided Drug Design*.

[13] Section of Interventional Cardiology of Chinese Society of Cardiology, Section of Atherosclerosis and Coronary Artery Disease of Chinese Society of Cardiology, Specialty Committee on Prevention and Treatment of Thrombosis of Chinese College of Cardiovascular Physicians (2018). Guideline on the diagnosis and treatment of stable coronary artery disease. *Chinese Journal of Cardiovascular Diseases*.

[14] Wan Z, Guo J, Pan A, Chen C, Liu L, Liu G (2021). Association of Serum 25-Hydroxyvitamin D Concentrations With All-Cause and Cause-Specific Mortality Among Individuals With Diabetes. *Diabetes Care*.

[15] Mancia G, Kreutz R, Brunström M, Burnier M, Grassi G, Januszewicz A (2023). 2023 ESH Guidelines for the management of arterial hypertension The Task Force for the management of arterial hypertension of the European Society of Hypertension: Endorsed by the International Society of Hypertension (ISH) and the European Renal Association (ERA). *Journal of Hypertension*.

[16] Johansen MC, Gottesman RF (2021). Cerebrovascular Disease and Cognitive Outcome in Patients with Cardiac Disease. *Seminars in Neurology*.

[17] Hsu JC, Yang YY, Chuang SL, Lin LY, Chen THH (2023). Prediabetes as a risk factor for new-onset atrial fibrillation: the propensity-score matching cohort analyzed using the Cox regression model coupled with the random survival forest. *Cardiovascular Diabetology*.

[18] Hou D, Zhang Y, Wu J, Li Y, An Z (2012). Reliability and validity of Chinese version of Barthel index. *Clinical Summary*.

[19] Sun Z, Liu X, Jiao L, Zhou T, Yang L, Fan J (2017). Study on the reliability and validity of Hospital Anxiety and Depression Scale. *Chinese Journal of Clinicians (Electronic Version)*.

[20] Dong L, Qiao X, Tian X, Liu N, Jin Y, Si H (2018). Cross-Cultural Adaptation and Validation of the FRAIL Scale in Chinese Community-Dwelling Older Adults. *Journal of The American Medical Directors Association*.

[21] Qu J, Zhou T, Xue M, Sun H, Shen Y, Chen Y (2021). Correlation Analysis of Hemoglobin-to-Red Blood Cell Distribution Width Ratio and Frailty in Elderly Patients With Coronary Heart Disease. *Frontiers in Cardiovascular Medicine*.

[22] Folsom AR, Boland LL, Cushman M, Heckbert SR, Rosamond WD, Walston JD (2007). Frailty and risk of venous thromboembolism in older adults. *The Journals of Gerontology. Series A, Biological Sciences and Medical Sciences*.

[23] Vermeulen J, Neyens JCL, van Rossum E, Spreeuwenberg MD, de Witte LP (2011). Predicting ADL disability in community-dwelling elderly people using physical frailty indicators: a systematic review. *BMC Geriatrics*.

[24] Gadó K, Khodier M, Virág A, Domján G, Dörnyei G (2022). Anemia of geriatric patients. *Physiology International*.

[25] Saraswat VA, Kumar K (2022). Untangling the Web of Malnutrition, Sarcopenia, and Frailty in Chronic Liver Disease. *Journal of Clinical and Experimental Hepatology*.

[26] Jayanama K, Theou O, Blodgett JM, Cahill L, Rockwood K (2018). Frailty, nutrition-related parameters, and mortality across the adult age spectrum. *BMC Medicine*.

[27] Yuan Y, Chen S, Lin C, Huang X, Lin S, Huang F (2023). Association of triglyceride-glucose index trajectory and frailty in urban older residents: evidence from the 10-year follow-up in a cohort study. *Cardiovascular Diabetology*.

[28] Li H, Zhao W (2023). Prevalence and influencing factors of frailty in elderly patients with chronic heart failure. *Journal of Chronic Diseases*.

[29] Bakhtiari M, Shaker F, Shirmard FO, Jalali A, Vakili-Basir A, Balabandian M (2024). Frailty efficacy as a predictor of clinical and cognitive complications in patients undergoing coronary artery bypass grafting: a prospective cohort study. *BMC Cardiovascular Disorders*.

[30] Chung CP, Chou KH, Chen WT, Liu LK, Lee WJ, Chen LK (2016). Cerebral microbleeds are associated with physical frailty: a community-based study. *Neurobiology of Aging*.

[31] Burton JK, Stewart J, Blair M, Oxley S, Wass A, Taylor-Rowan M (2022). Prevalence and implications of frailty in acute stroke: systematic review & meta-analysis. *Age and Ageing*.

[32] Bu F, Deng XH, Zhan NN, Cheng H, Wang ZL, Tang L (2023). Development and validation of a risk prediction model for frailty in patients with diabetes. *BMC Geriatrics*.

[33] Fujii K, Fujii Y, Kitano N, Sato A, Hotta K, Okura T (2021). Mediating role of instrumental activities of daily living ability on cognitive function of older adults living alone: A 4-year longitudinal study from the Kasama study. *Medicine*.

[34] Wang X, Hu J, Wu D (2022). Risk factors for frailty in older adults. *Medicine*.

[35] Yanagita I, Fujihara Y, Eda T, Tajima M, Yonemura K, Kawajiri T (2018). Low glycated hemoglobin level is associated with severity of frailty in Japanese elderly diabetes patients. *Journal of Diabetes Investigation*.

[36] Liu S, Yuan X, Liang H, Jiang Z, Yang X, Gao H (2024). Development and validation of frailty risk prediction model for elderly patients with coronary heart disease. *BMC Geriatrics*.

